# Post-oncologic skin health: cutaneous recovery and rehabilitation after cancer treatments

**DOI:** 10.3389/fonc.2026.1774381

**Published:** 2026-03-11

**Authors:** Diala Haykal, Anthony Rossi, Delphine Kerob, Brigitte Dréno

**Affiliations:** 1 Centre Laser Palaiseau, Private Practice, Palaiseau, France; 2 Dermatology Service, Department of Medicine, Memorial Sloan Kettering Cancer Center, New York, NY, United States; 3 Oncodermatology Department, Saint Louis Hospital, Paris, France; 4 Nantes Université, INSERM, CNRS, Immunology and New Concepts in Immuno Therapy, INCIT, UMR, Nantes, France

**Keywords:** cancer survivorship, cutaneous toxicities, microbiome modulation, photobiomodulation, post-oncologic dermatology, radiation dermatitis, regenerative dermatology, skin barrier repair

## Abstract

**Background:**

Advances in oncology have markedly improved cancer survival, however, chronic cutaneous sequelae induced by chemotherapy, radiotherapy, targeted therapies, and immunotherapies remain common and may persist long after treatment completion. These long-term skin changes, ranging from xerosis and pruritus to pigmentary alterations, fibrosis, and immune-mediated dermatoses, can substantially impair quality of life and functional recovery.

**Objective:**

To define the evolving role of dermatology in post-oncologic skin health and to critically synthesize mechanistic and clinical evidence supporting long-term dermatologic rehabilitation strategies.

**Methods:**

A structured search of PubMed and MEDLINE (2010–2025) identified clinical and mechanistic studies, consensus guidelines, systematic reviews, and cohort data relevant to post-treatment cutaneous sequelae. Eligible publications included human studies addressing chronic skin changes after cancer therapy or evaluating therapeutic approaches applicable to survivorship dermatology. The search yielded 612 records, of which 148 full-text articles were reviewed and 54 met the inclusion criteria for synthesis.

**Results:**

Post-oncologic skin care extends beyond symptomatic management to encompass restoration of epidermal barrier function, mitigation of chronic inflammation, support of the cutaneous microbiome, and improvement of psychosocial well-being. Mechanistic studies highlight persistent structural, immunologic, molecular, and microbial alterations that underline delayed skin recovery. Within this context, photobiomodulation and selective energy-based modalities may support long-term cutaneous resilience, however robust clinical evidence of their benefit is lacking.

**Conclusion:**

Dermatologists play a central role in survivorship care by transitioning cutaneous management from symptomatic treatment toward preventive, restorative, and rehabilitative strategies that improve long-term quality of life.

## 1 Introduction

**Figure 1:**
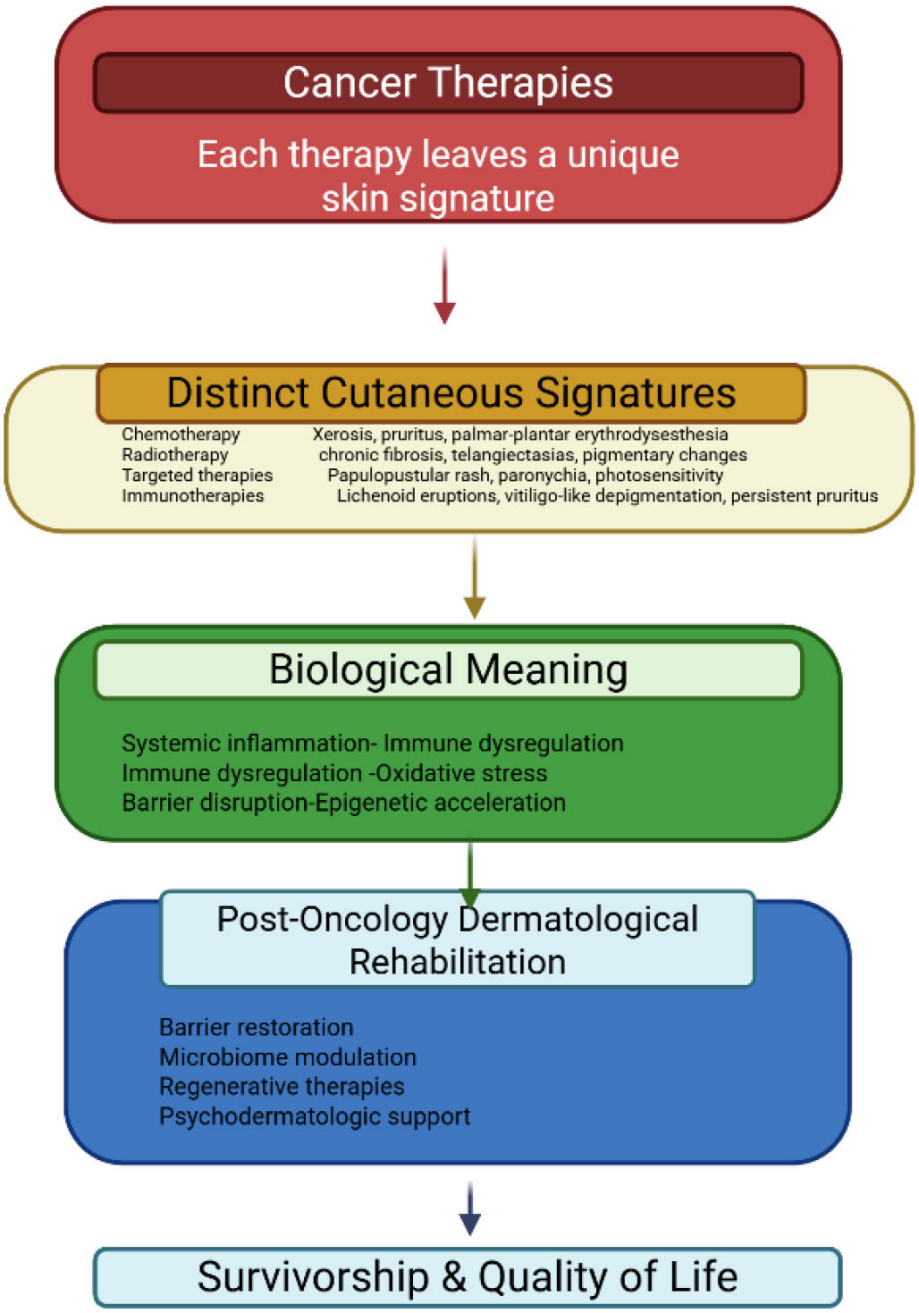
From cancer therapy to skin recovery.

Cutaneous toxicities are among the most visible consequences of cancer therapy. For many survivors, the end of oncologic treatment marks the beginning of a new set of dermatologic challenges, including persistent xerosis, pigmentary alterations, chronic pruritus, paronychia, post-radiation fibrosis, and immune-related dermatoses. These effects may compromise comfort, interfere with daily functioning, and influence psychosocial well-being long after treatment completion. For many survivors, these visible skin changes function as lasting reminders of illness, influencing daily comfort, social interactions, and self-perception long after cancer remission. As survivorship grows, dermatologists are increasingly called upon to address not only acute adverse events but also long-term skin rehabilitation. The goal extends beyond treating lesions to restoring barrier integrity, improving functional outcomes, and supporting the recovery of self-confidence and normalcy (**Figure 1**) (1–3). This review proposes a mechanism-based framework for post-oncologic skin rehabilitation, linking treatment-specific injuries to persistent skin phenotypes and targeted restorative strategies, while explicitly identifying current evidence gaps and research priorities.

## 2 Methods

A structured literature search was performed using PubMed and MEDLINE to identify publications relevant to post-oncologic cutaneous health. The search covered the period from January 2010 to October 2025 and used combinations of the following terms “cancer survivors, cutaneous toxicity, radiation dermatitis, chemotherapy-related skin effects, targeted therapy skin reactions, immune checkpoint inhibitor dermatologic toxicity, cutaneous sequelae, skin barrier repair, pigmentary alterations, photobiomodulation, microbiome, and post-oncologic skin care”. The database search initially retrieved 612 records, of which 148 full-text articles were assessed for eligibility. 54 were included in this review.

Studies were eligible for inclusion if they involved adult human cancer patients or survivors and provided clinical or mechanistic data on cutaneous effects of chemotherapy, radiotherapy, targeted therapies, or immunotherapies. Eligible designs included randomized or non-randomized clinical trials, prospective and retrospective cohort studies, case–control studies, large case series, consensus guidelines, systematic reviews, and meta-analyses. Publications were further considered if they reported histologic, immunologic, molecular, microbiome-related, or epigenetic changes in post-treatment skin, or if they described therapeutic approaches relevant to dermatologic rehabilitation such as barrier repair, dermocosmetics, photobiomodulation, or energy-based interventions. Studies were excluded if they focused exclusively on pediatric populations, reported only acute treatment reactions without follow-up, consisted of small case reports with limited relevance, addressed non-cutaneous toxicities, or lacked peer review or adequate methodological detail. After applying inclusion and exclusion criteria, 49 publications were ultimately included in this synthesis.

Titles and abstracts were screened to assess relevance, and full-text articles were reviewed when eligibility was uncertain. Extracted data included the type and timing of cutaneous sequelae, mechanistic insights into post-treatment skin biology, and evidence supporting therapeutic or preventive strategies. This methodological approach prioritizes translational relevance for survivorship-oriented dermatologic care.

## 3 Results

Cancer treatments leave characteristic dermatologic patterns that reflect underlying cellular and immunologic disruption. Chemotherapy often results in marked xerosis, impaired barrier function, palmar-plantar erythrodysesthesia, and nail fragility due to reduced keratinocyte turnover and sebaceous gland dysfunction. Radiotherapy induces acute erythema and desquamation, but the chronic phase may include telangiectasias, pigmentary changes, fibrosis, and atrophy resulting from fibroblast activation and endothelial injury. Targeted agents, including Epidermal growth factor receptor (EGFR), B-Raf proto-oncogene, serine/threonine kinase (BRAF), and Vascular endothelial growth factor (VEGF) inhibitors, produce predictable eruptions such as papulopustular rashes, paronychia, and photosensitivity, linked to the disruption of specific signaling pathways involved in epidermal and follicular homeostasis. Immunotherapies, particularly checkpoint inhibitors, can provoke a spectrum of immune-related cutaneous events ranging from lichenoid dermatitis to vitiligo-like depigmentation and chronic pruritus. These manifestations provide insight into systemic immune activation and may, in some cases, correlate with treatment response. Together, these diverse effects highlight the need for dermatologic rehabilitation long after oncologic care has ended (4–7). Findings are presented according to treatment modality and timing of cutaneous sequelae in order to clarify differences in pathophysiology, persistence, and strength of supporting evidence.

### 3.1 Cutaneous sequelae of cancer therapy

Cancer therapies produce a diverse range of acute and chronic cutaneous manifestations, many of which persist long after treatment has concluded. These sequelae arise from disruptions in keratinocyte dynamics, adnexal function, microvascular integrity, and immune regulation. Because skin is one of the most visible interfaces between the patient and their treatment history, these chronic changes often affect comfort, appearance, and psychosocial well-being. A clear understanding of treatment-specific patterns is essential for structuring effective post-oncologic dermatologic rehabilitation.

### 3.2 Chemotherapy-related sequelae

Chemotherapeutic agents rapidly dividing epidermal and adnexal cells, leading to significant and long-lasting changes in skin physiology. Patients commonly develop pronounced xerosis characterized by dryness, rough texture, fissuring, and heightened sensitivity due to diminished lipid synthesis and delayed keratinocyte turnover. Chronic pruritus is also frequent and may persist long after treatment, reflecting low-grade inflammation, sensory nerve hypersensitivity, and microbiome alterations (8, 9). Pigmentary disturbances such as diffuse hyperpigmentation, serpiginous venous patterns, or drug-specific patterns also occur. Nail involvement is especially common, with onycholysis, ridging, brittleness, and paronychia developing from matrix toxicity. Collectively, these manifestations illustrate the broad and sustained impact of cytotoxic agents on epidermal homeostasis (10). These studies indicate that xerosis, pruritus, and nail dystrophy may persist for months to years after chemotherapy completion, underscoring the need for long-term barrier-focused care.

### 3.3 Radiation-induced sequelae

Radiotherapy elicits a well-described continuum of skin injury, progressing from acute inflammation to chronic structural remodeling. While the acute phase typically presents with erythema, edema, and desquamation, the chronic phase is characterized by more persistent vascular and fibrotic alterations (11). Telangiectasias result from endothelial injury and aberrant microvascular repair. Fibrosis and induration reflect prolonged fibroblast activation and collagen deposition. Additional long-term effects include atrophy, reduced elasticity, xerosis from adnexal gland destruction, and pigmentary changes that may be either hyper- or hypopigmented. Radiation-altered skin is biomechanically fragile, slower to heal, and often less tolerant to cosmetic or medical procedures, making it a critical focus of post-treatment rehabilitation (12). These late effects often render irradiated skin less tolerant to procedural interventions and slower to heal, necessitating long-term dermatologic surveillance.

### 3.4 Endocrine therapy–associated sequelae

Endocrine therapies are central to the long-term management of hormone-sensitive malignancies and are frequently continued for years. Unlike cytotoxic treatments, their dermatologic impact reflects sustained hormonal deprivation rather than direct cellular injury. Estrogen and androgen signaling play key roles in maintaining epidermal barrier integrity, sebaceous function, dermal collagen homeostasis, and hair follicle cycling. As a result, endocrine therapy may be associated with progressive xerosis, increased cutaneous sensitivity, pruritus, and features resembling accelerated cutaneous aging, including reduced elasticity and impaired reparative capacity (13, 14).

Hair changes are among the most distressing sequelae in survivorship. Endocrine-induced alopecia typically presents as diffuse thinning or a female-pattern hair loss–like distribution and may persist throughout maintenance therapy, contributing substantially to psychosocial burden and altered self-image (15, 16).

Exploratory use of autologous biologics such as platelet-rich plasma (PRP) has been reported for endocrine-induced alopecia (EIA) and persistent chemotherapy-induced alopecia (pCIA) in breast cancer survivors. In a randomized controlled pilot study by Rossi et al. (n = 27; 15 EIA, 12 pCIA), global assessment scores improved from baseline to week 12 in both groups; however, improvements were not significantly different between PRP-treated and untreated sides. Hair density increased on both sides without a between-side difference, and quality-of-life scores did not significantly improve. Adverse events were mainly scalp pain (grade 1–3). Notably, circulating tumor cells were detected in 2 of 12 assays performed on PRP samples, with no tumor seeding events observed during the study period—highlighting the need for careful oncologic risk assessment, standardized processing, and shared decision-making when considering PRP in survivorship settings (17).

### 3.5 Targeted therapy–associated sequelae

Targeted oncologic agents provoke distinct and often predictable cutaneous patterns due to their interference with specific signaling pathways involved in epidermal and follicular biology. EGFR inhibitors commonly induce papulopustular eruptions, alongside marked xerosis, fissuring, and periungual inflammation. Inhibitors of the BRAF and Mitogen-Activated Protein Kinase/Extracellular Signal–Regulated Kinase Kinase (MEK) pathways may lead to photosensitivity, eruptive nevi, keratoses, or granulomatous reactions, reflecting the pathways’ roles in melanocyte and keratinocyte regulation. VEGF inhibitors impair angiogenesis and vascular repair, contributing to delayed wound healing and increased susceptibility to ecchymoses (18, 19).

### 3.6 Immunotherapy-related sequelae

Immune checkpoint inhibitors produce a wide spectrum of immune-mediated cutaneous reactions that can arise during treatment or appear months later. Lichenoid dermatitis, eczematous or psoriasiform eruptions, and chronic pruritus, affecting up to half of patients, are among the most frequently observed manifestations. Vitiligo-like depigmentation, particularly common in melanoma patients, reflects heightened T-cell activity and may correlate with positive therapeutic response. These chronic immune-related sequelae often persist even after therapy cessation and may require sustained dermatologic management. Their presence highlights the complex interplay between systemic immune activation and cutaneous homeostasis (20, 21).

### 3.7 Long-term and late-onset sequelae

Across all treatment modalities, many survivors experience persistent dermatologic issues that evolve over time (22). Chronic xerosis, barrier fragility, pigmentary irregularities, neuropathic or inflammatory pruritus, nail dystrophy, and alopecia may continue for months or years. Radiation-induced fibrosis and changes in skin texture can limit mobility and functional comfort, while oxidative stress and treatment-induced epigenetic alterations may contribute to accelerated cutaneous aging. The cumulative burden of these late sequelae may contribute to functional limitation, accelerated cutaneous aging, and sustained psychosocial distress.

### 3.8 Post-oncologic skin health

Cancer survivors frequently experience persistent cutaneous changes that extend long after the completion of therapy, making skin recovery a central component of survivorship medicine. Post-oncologic skin health therefore requires a structured, multidimensional approach that addresses barrier repair, restorative and regenerative interventions, psychosocial well-being, preventive dermatology, and the biological mechanisms that shape long-term healing. Emerging technologies, including digital monitoring and AI-assisted support, further enhance follow-up and personalization of care. This framework shifts dermatologic care from reactive symptom control toward anticipatory and preventive survivorship management.

#### 3.8.1 Dermatologic rehabilitation in survivorship

Post-oncologic dermatologic care has evolved from a palliative model to one oriented toward functional recovery and long-term skin health. Central to this approach is the restoration of the epidermal barrier, which is commonly impaired across treatment modalities. Ceramide-rich moisturizers, niacinamide-containing formulations, humectants such as glycerin, and gentle emollients can significantly reduce transepidermal water loss and improve tolerance (23). Products incorporating postbiotic ingredients, such as Vitreoscilla filiformis extract or probiotic lysates, may help re-establish microbial balance and enhance epidermal immunity. Establishing a consistent, structured routine is essential for mitigating xerosis, reducing pruritus, and preparing the skin for more advanced interventions when needed (23–25).

Microbiome-focused skincare represents an emerging area in post-oncologic rehabilitation. Cancer therapies often provoke dysbiosis, resulting in heightened sensitivity and delayed recovery. Although dedicated oncology trials remain limited, mounting evidence suggests that supporting commensal microorganisms can improve barrier integrity, reduce inflammation, and increase cutaneous resilience. Microbiome-friendly cleansers, minimal-ingredient emollients, and postbiotic-rich formulations may complement traditional supportive care (26, 27).

### 3.9 Therapeutic approaches in post-oncologic skin health

#### 3.9.1 Cosmetic and dermocosmetic therapies

Cosmetic and dermocosmetic care forms the foundation of post-oncologic rehabilitation. Cancer treatments commonly impair barrier function, disrupt microbiomes, and increase sensitivity, making gentle but targeted skincare essential. Ceramide-rich moisturizers, niacinamide-containing products, humectants such as glycerin, and lipid-replenishing emollients help reduce transepidermal water loss and restore cutaneous integrity (23, 28). Postbiotic and microbiome-supportive formulations may further improve tolerance and resilience by rebalancing dysbiosis induced by therapy. A consistent, structured routine enhances comfort, reduces pruritus, and prepares the skin for more advanced interventions (29). Products that are also devoid of known contact sensitizers, allergens, and hormone disruptors are particularly important for patients with cancer.

#### 3.9.2 Energy-based and regenerative interventions

Interest has been raised in the potential use of energy-based and biologic approaches as part of post-oncologic skin care; however, current clinical evidence in oncology and cancer survivorship populations remains limited. Non-ablative energy-based devices and photobiomodulation have been described in supportive dermatologic settings, primarily based on mechanistic data and extrapolation from non-oncologic indications (**Table 1**) (31). In the absence of robust clinical trials demonstrating clear benefit and safety in cancer survivors, these modalities should currently not be recommended. Similarly, biologic approaches such as PRP has shown no significant benefit in endocrine-induced alopecia or persistent chemotherapy-induced alopecia should only be considered in highly selected cases, following multidisciplinary discussion, explicit patient information, and with oncologic safety as the primary consideration (17).

**Table 1:** Evidence-based applications of photobiomodulation in oncologic and post-oncologic dermatology (30).

Dermatologic condition treated with PhotoBiomodulation	Level of evidence
Conditions secondary to cancer therapies	
Mucositis	IA
Acute radiation dermatitis	IA
Lymphedema	IA

#### 3.9.3 Psychodermatologic considerations

The dermatologic consequences of cancer therapy often extend beyond physical discomfort to influence identity, social confidence, and emotional well-being. Pigmentary alterations, alopecia, scarring, and nail changes may serve as visible reminders of illness (22, 32). Dermatologists are well positioned not only to treat these conditions but also to help patients navigate the psychological dimensions of their recovery. Safe aesthetic interventions, such as pigment correction, gentle resurfacing, or hyaluronic acid-based fillers, would play a meaningful role in helping survivors regain a sense of normalcy and control over their appearance. Addressing these concerns is integral to survivorship care, as cutaneous appearance often plays a central role in identity reconstruction after cancer (33).

#### 3.9.4 Preventive dermatology across the treatment continuum

Preventive strategies are essential throughout the oncologic journey and significantly influence long-term outcomes. Dermatologic assessment before therapy allows clinicians to identify pre-existing conditions such as eczema or rosacea that could be amplified during treatment and to establish individualized routines emphasizing gentle cleansing, adequate moisturization, and rigorous photoprotection (34, 35). During cancer therapy, early recognition and management of eruptions, mucositis, or paronychia help maintain treatment adherence and reduce complications. After therapy, long-term surveillance is critical for detecting chronic radiation effects, persistent or late-onset immune-related conditions, and secondary skin malignancies. Preventive dermatology should be implemented across the continuum of care, before, during, and after cancer treatment, to mitigate both acute and chronic sequelae (36–38).

### 3.10 Biological mechanisms underlying cutaneous recovery

Cutaneous recovery after cancer therapy reflects a multifactorial process involving persistent structural, immunologic, molecular, and microbial alterations. Histologically, chemotherapeutic agents have been shown to reduce keratinocyte proliferation, thin the epidermis, and cause adnexal atrophy, contributing to lasting xerosis and barrier fragility. In radiotherapy fields, chronic microvascular injury, persistent fibroblast activation, and extracellular matrix remodeling have been documented in long-term human biopsy studies, correlating with clinical telangiectasias, fibrosis, and dermal atrophy. These structural abnormalities are considered major contributors to impaired healing and reduced biomechanical resilience in previously treated skin (39).

From an immunologic standpoint, cytotoxic chemotherapy diminishes cutaneous immune surveillance, including reduced numbers and impaired function of Langerhans cells, while immune checkpoint inhibitors provoke an opposite pattern of heightened T-cell activation and immune dysregulation. These shifts in immune balance underline the chronic inflammatory states observed in cancer survivors and contribute to long-term pruritus, xerosis, and immune-mediated dermatoses. In irradiated skin, sustained expression of pro-fibrotic cytokines such as TGF-β has been demonstrated, supporting its role in the chronic evolution of dermal thickening and fibrosis (40, 41).

Transcriptomic studies, although limited to small human cohorts, consistently demonstrate upregulation of oxidative stress pathways, DNA damage responses, and mitochondrial stress signatures after chemotherapy and radiotherapy. These molecular changes occur alongside downregulation of genes involved in barrier function, lipid metabolism, and keratinocyte differentiation, collectively contributing to delayed epidermal repair and prolonged sensitivity. Pro-fibrotic transcriptomic programs, including increased expression of COL1A1, COL3A1, and ACTA2, have been identified in irradiated skin months to years after treatment, reinforcing clinical observations of long-lasting dermal remodeling (42, 43).

Alterations of the skin microbiome have also been reported in patients undergoing cancer therapy. Human studies show reductions in microbial diversity and loss of key commensals such as Cutibacterium acnes and Staphylococcus epidermidis during chemotherapy, as well as shifts toward pro-inflammatory microbial communities in therapy-induced dermatitis. These dysbiotic patterns impair innate immune function, hinder epidermal repair, and contribute to increased sensitivity during recovery. While the magnitude of microbiome disruption varies, the directionality of these changes is consistently demonstrated across available studies (44).

Oxidative stress represents another central biological driver of long-term cutaneous sequelae. Chemotherapy and radiotherapy both induce mitochondrial dysfunction, increase reactive oxygen species, and impair cellular energy metabolism, leading to reduced keratinocyte migration and fibroblast-mediated matrix repair (45, 46). Photobiomodulation has been shown in mechanistic and early clinical studies to improve mitochondrial activity, attenuate oxidative stress, and modulate inflammatory responses, thereby suggesting potential benefit for fragile, treatment-affected skin; however, clinical validation in post-oncologic populations remains limited (31, 47).

Beyond inflammatory and oxidative pathways, emerging research shows that cancer therapies exert durable epigenetic effects. Changes in DNA methylation, chromatin accessibility, and histone modifications have been observed in skin cells after chemotherapy and radiotherapy, suggesting accelerated epigenetic aging and persistent downregulation of genes involved in barrier homeostasis and antioxidant defense. These epigenetic imprints may contribute to the prolonged vulnerability, dryness, and delayed repair characteristic of post-oncologic skin (48).

Together, these histologic, immunologic, transcriptomic, microbiome-related, oxidative, and epigenetic mechanisms provide a biologically coherent explanation for the slow and sometimes incomplete cutaneous recovery observed in cancer survivors. They also underscore the importance of integrating barrier repair, microbiome-supportive skincare, anti-inflammatory measures, and regenerative therapies into survivorship-oriented dermatologic care.

### 3.11 Digital and AI-assisted support

Artificial intelligence is becoming an increasingly valuable component of post-oncologic dermatology. Digital imaging and teledermatology platforms allow for standardized, remote monitoring of erythema, xerosis, fibrosis, pigmentary alterations, and other chronic skin changes that may evolve months after treatment completion. These tools enable earlier recognition of subtle deterioration in barrier function or inflammatory activity and facilitate timely adjustments to skincare regimens or therapeutic interventions (49, 50). However, most currently available systems rely on heterogeneous datasets and lack prospective validation in cancer survivor populations, with limited representation of diverse phototypes, complex treatment regimens, and late-onset sequelae. Machine-learning models are being developed to integrate oncologic treatment history, skin phenotype, genetic predisposition, and environmental factors to identify individuals at higher risk of persistent or late-onset cutaneous toxicities; nonetheless, challenges related to algorithmic bias, limited interpretability, and real-world clinical integration remain significant. At present, AI-assisted tools should therefore be considered adjunctive decision-support technologies rather than standalone diagnostic or predictive systems. Despite these limitations, AI-enabled platforms may enhance continuity of care, expand access to dermatologic expertise in underserved settings, and support more proactive, personalized skin health management for cancer survivors (51).

### 3.12 Clinical take-home

Early dermatologic involvement throughout the oncologic care continuum can substantially improve both treatment tolerance and long-term skin health. Integrating dermatologists at the initiation of therapy enables anticipatory guidance, baseline assessment of pre-existing vulnerabilities, and early management of emerging toxicities, interventions that have been associated with improved adherence to systemic therapies and reduced need for dose modifications. Dermatologic management of radiation dermatitis, targeted-therapy eruptions, or immune-related cutaneous adverse events help preserve oncologic treatment plans while minimizing discomfort, functional impairment, and psychosocial burden. This is particularly important for prolonged radiotherapy courses or multimodal regimens, in which cumulative skin injury may otherwise lead to treatment interruptions. Incorporating structured barrier repair, photoprotection, microbiome-supportive care, and psychosocial support into survivorship pathways contributes to improved quality of life and sustained cutaneous resilience long after treatment ends. Integrating dermatology into survivorship pathways improves both cutaneous outcomes and overall quality of life (52).

## 4 Conclusion

Post-oncologic skin recovery is shaped by persistent structural, immunologic, molecular, and microbiome-related alterations that can continue long after cancer treatment has ended. These changes impair barrier integrity, delay repair, and contribute to chronic symptoms that significantly affect quality of life. As survivorship becomes an increasingly prominent phase of cancer care, dermatologists play a critical role in restoring cutaneous health, preventing long-term sequelae, and supporting psychosocial well-being. Early dermatologic involvement throughout the oncologic care continuum can substantially improve both treatment tolerance and long-term skin outcomes. Integrating dermatologists at the initiation of therapy enables anticipatory guidance, identification of pre-existing vulnerabilities, and early management of emerging toxicities.

Incorporating structured barrier repair, photoprotection, microbiome-supportive care, anti-inflammatory strategies, and psychosocial support into survivorship pathways enhances quality of life and promotes durable cutaneous resilience. Growing evidence confirms dermatology’s central role in optimizing survivorship outcomes and guiding patients through the transition from active therapy to long-term recovery. Strengthening collaboration between oncologists and dermatologists is therefore essential for ensuring that cancer survivors not only live longer but live with healthier skin and improved overall well-being.
